# Identification of strong intron enhancer in the heparanase gene: effect of functional rs4693608 variant on HPSE enhancer activity in hematological and solid malignancies

**DOI:** 10.1038/s41389-018-0060-8

**Published:** 2018-06-29

**Authors:** Olga Ostrovsky, Ania Hava Grushchenko-Polaq, Katia Beider, Margarita Mayorov, Jonathan Canaani, Avichai Shimoni, Israel Vlodavsky, Arnon Nagler

**Affiliations:** 1 Department of Hematology and Bone Marrow Transplantation, Chaim Sheba Medical Center, Tel-Hashomer, Israel; 2 Cancer and Vascular Biology Research Center, Rappaport Faculty of Medicine, Technion, Haifa, Israel

## Abstract

Heparanase is an endo-β-glucuronidase that specifically cleaves the saccharide chains of heparan sulfate (HS) proteoglycans and releases HS-bound cytokines, chemokines, and bioactive growth-promoting factors. Heparanase plays an important role in the nucleus as part of an active chromatin complex. Our previous studies revealed that rs4693608 correlates with heparanase levels and increased risk of acute and extensive chronic graft vs. host disease (GVHD). Discrepancy between recipient and donor in this SNP significantly affected the risk of acute GVHD. In the present study, we analyzed the HPSE gene region, including rs4693608, and demonstrated that this region exhibits SNPs-dependent enhancer activity. Analysis of nuclear proteins from normal leukocytes revealed their binding to DNA probe of both alleles with higher affinity to allele G. All malignant cell lines and leukemia samples disclosed a shift of the main bands in comparison to normal leukocytes. At least five additional shifted bands were bound to allele A while allele G probe was bound to only one main DNA/protein complex. Additional SNPs rs4693083, rs4693084, and rs4693609 were found in strong linkage disequilibrium (LD) with rs11099592 (exon 7). Only rs4693084 affected protein binding to DNA in cell lines and leukemia samples. As a result of the short distance between rs4693608 and rs4693084, both SNPs may be included in a common DNA/protein complex. DNA pull-down assay revealed that heparanase is involved in self-regulation by negative feedback in rs4693608-dependent manner. During carcinogenesis, heparanase self-regulation is discontinued and the helicase-like transcription factor begins to regulate this enhancer region. Altogether, our study elucidates conceivable mechanism(s) by which rs4693608 SNP regulates HPSE gene expression and the associated disease outcome.

## Introduction

Heparanase is an endo-β-glucuronidase that specifically cleaves the saccharide chains of heparan sulfate (HS) proteoglycans (HSPG), important components of the cell surface, basement membrane, and extracellular matrix (ECM). Cleavage of HS by active heparanase leads to loss of integrity of the basement membrane and ECM and release of HS-bound cytokines, chemokines, and growth-promoting factors^1–6^.

Heparanase plays critical roles in tumor metastasis and angiogenesis^5^. High expression of heparanase is frequently observed in an increasing number of primary human tumors of all etiologies (carcinoma, sarcoma, and hematological malignancies), correlating with high vessel density and poor clinical outcome^7–10^. The enzyme is also involved in inflammation^11^, fibrosis^12^, diabetes^13^, and kidney dysfunction^14^.

Heparanase can trigger the phosphorylation of protein kinases, such as p38, Erk, and Akt, a process mediated by the C-terminal domain of the protein^5,15^. Moreover, heparanase plays a role in the nucleus as part of an active chromatin complex which regulates inducible gene transcription. Nuclear heparanase associates with euchromatin and regulates histone H3K4 and H3K9 methylation by binding to target gene regulatory regions in association with the demethylase LSD1^16^.

A number of functional single-nucleotide polymorphisms (SNPs) were identified in the HPSE gene^17–19^, rs4693608 polymorphism being the most prominent^19,20^. Combinations of rs4693608 and rs4364254 were found in significant correlation with heparanase levels in normal leukocytes and both before and after pre-transplantation conditioning^19,21^. In addition, a highly significant association was identified between rs4693608 and the risk of acute and extensive chronic graft vs. host disease (GVHD). Moreover, discrepancy between recipient and donor in this SNP significantly affected the risk of GVHD^20^. A positive association was found between recipients and donors rs4693608 discrepancy and the recovery time of neutrophils and platelets^21^. rs4693608 also affects HPSE gene expression in lipopolysaccharide (LPS)-treated mononuclear cells (MNC) from peripheral and cord blood^21^.

Recently, rs4693608 and rs4364254 were found to be a genetic risk factor for sinusoid obstruction syndrome/veno-occlusive disease^22^. These polymorphisms were also associated with different levels of HPSE mRNA within carotid plaque tissue^23^. Functional HPSE SNPs were found to play a role in gastric^24,25^ and ovarian^26^ cancer progression and patient survival. In addition, rs4693608 and rs6535455 correlate with bone disease and the outcome of multiple myeloma patients^27^.

In the present study, we analyzed 440 bp of the HPSE gene (intron 2) (chr4: 84,241,177-84,242,376) that include the most prominent rs4693608 SNP. Our results indicate that this region exhibits enhancer activity in both the sense and antisense directions. A–G alteration leads to a different enhancer activity as a result of a unique DNA–protein complex, which binds to DNA regulatory elements in the enhancer. The DNA–protein complex that binds to the G allele shifted similarly in all the examined cell lines and primary leukemic samples, but not in normal white blood cells. At least five different variations of DNA–protein complexes bound to allele A were identified. DNA pull-down assay of normal cell samples revealed that heparanase is involved in self-regulation of the HPSE gene by negative feedback in rs4693608-dependent manner. During carcinogenesis, heparanase self-regulation is attenuated and the helicase-like transcription factor begins to regulate this enhancer region. Altogether, the present study reveals points to a possible mechanism by which rs4693608 affects HPSE gene expression and thereby dictates disease outcome.

## Results

### Characterization of SNPs in the cloned enhancer region

**Fig. 1:**
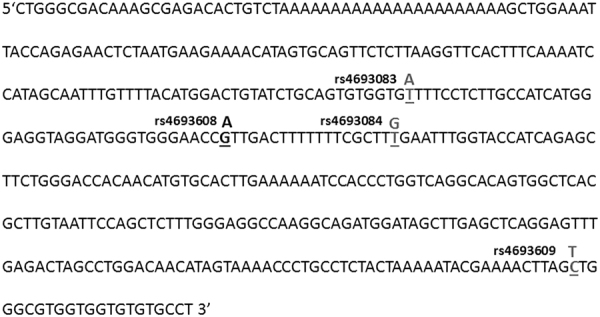
Map of the HPSE enhancer region. The size of analyzed enhancer region is 440 bp. It includes four polymorphic SNPs: rs4693083, rs4693608, rs4693084, and rs4693609. The map represents exact location of the SNPs and nucleotide substitution. The distance between rs4693083 and rs4693608 is 40 bp, whereas the interval between rs4693608 and rs4693084 is 17 bp. Additional rs4693609 SNP is located 199 bp upstream of rs4693084

According to the UCSC Genome Browser website, the region that contains rs4693608 SNP is a putative strong HPSE enhancer. Detailed analysis of the enhancer region revealed additional three polymorphic SNPs, which were mapped in this area (Fig. 1). As shown in Fig. 1, the distance between rs4693083 and rs4693608 is 40 bp, whereas the interval between rs4693608 and rs4693084 is 17 bp. Additional rs4693609 SNP is located 199 bp upstream the rs4693084 (Fig. 1). Genotyping of the SNPs was performed among 148 healthy individuals and the results are summarized in Table 1. The SNP genotype frequencies did not significantly deviate from the Hardy–Weinberg distribution (data not shown). rs4693083, rs4693084, and rs4693609 were found in strong linkage disequilibrium (LD). Only three out of the 148 healthy individuals had a different genotype for rs4693609 SNP in comparison to rs4693083 and rs4693084 polymorphisms. However, all three SNPs exhibited a partial LD with the functional rs4693608 SNP. In addition, we found that rs11099592, located in exon 7, is in strong LD with each of the three SNPs (Table 1).

**Table 1:** Genotype and allele frequencies of the HPSE gene SNPs

SNPs	Position	Genotypes and alleles	No	Frequencies, %
rs4693084	Intron 3	GG	91	61.5
		GT	50	33.8
		TT	7	4.7
		G	232	78.4
		T	64	21.6
rs4693083	Intron 3	AA	91	61.5
		AT	50	33.8
		TT	7	4.7
		A	232	78.4
		T	64	21.6
rs4693609	Intron 3	TT	88	59.5
		TC	53	35.8
		CC	7	4.7
		T	229	77.4
		C	67	22.6
rs4693608	Intron 3	AA	56	37.8
		AG	62	41.9
		GG	30	20.3
		A	174	58.8
		G	122	41.2
rs11099592	Exon 7	GG	91	61.5
		GA	49	33.1
		AA	8	5.4
		G	231	78
		A	65	22

Our previous study identified the presence of two blocks of SNPs in the HPSE gene^16^. The first block includes polymorphisms which were mapped between intron 1 and exon 7. The second block starts at rs6856901 (3′UTR). However, the most significant rs4693608 was found in partial LD with all analyzed SNPs in spite of the presence of two blocks. rs4693083, rs4693084, and rs4693609 are located in the first block and were found in strong LD with rs11099592 (exon 7). Hence, these SNPs are in complete LD with rs6535455 (intron 4) and rs11099594 (intron 2, 510 bp downstream of rs4693608), which were previously found in strong LD with rs11099592^19^. Previous studies revealed an association between rs11099592 and the risk of acute lymphoblastic leukemia (ALL) development^22^, and poor outcome of gastric cancer^24,25^. As a result of strong LD between rs11099592 and the other SNPs located in introns 2 and 4, we assume that newly identified additional SNPs may also correlate with the malignant phenotype of these cancers.

### Detection of enhancer activity in hematological and non-hematological cancer cell lines

**Fig. 2:**
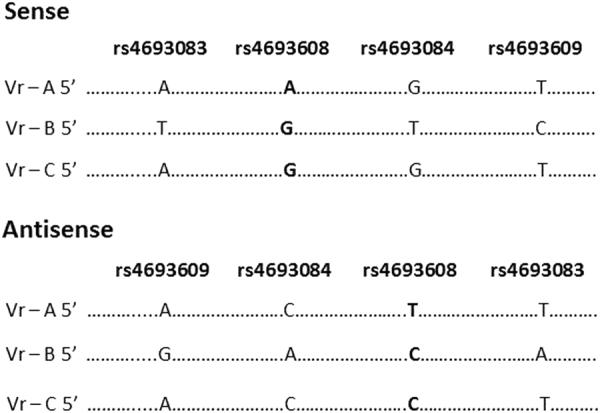
Structure of six DNA constructs for luciferase reporter assay. The HPSE gene fragment of intron 2 was cloned via PCR using PCR II-TOPO vector. The enhancer fragments were digested with *Hin*dIII and *Xho*I and ligated into phosphatase-treated pGL4.26 (luc2/minP/Hygro) vector that was digested with the same enzymes. Six DNA constructs were prepared, three in the sense direction and three in the antisense direction. The constructs were named variant (Vr) A, B, and C, according to the linkage disequilibrium (LD) between SNPs and possibility of allele variants. SNP rs4693608 is marked in bold

**Fig. 3:**
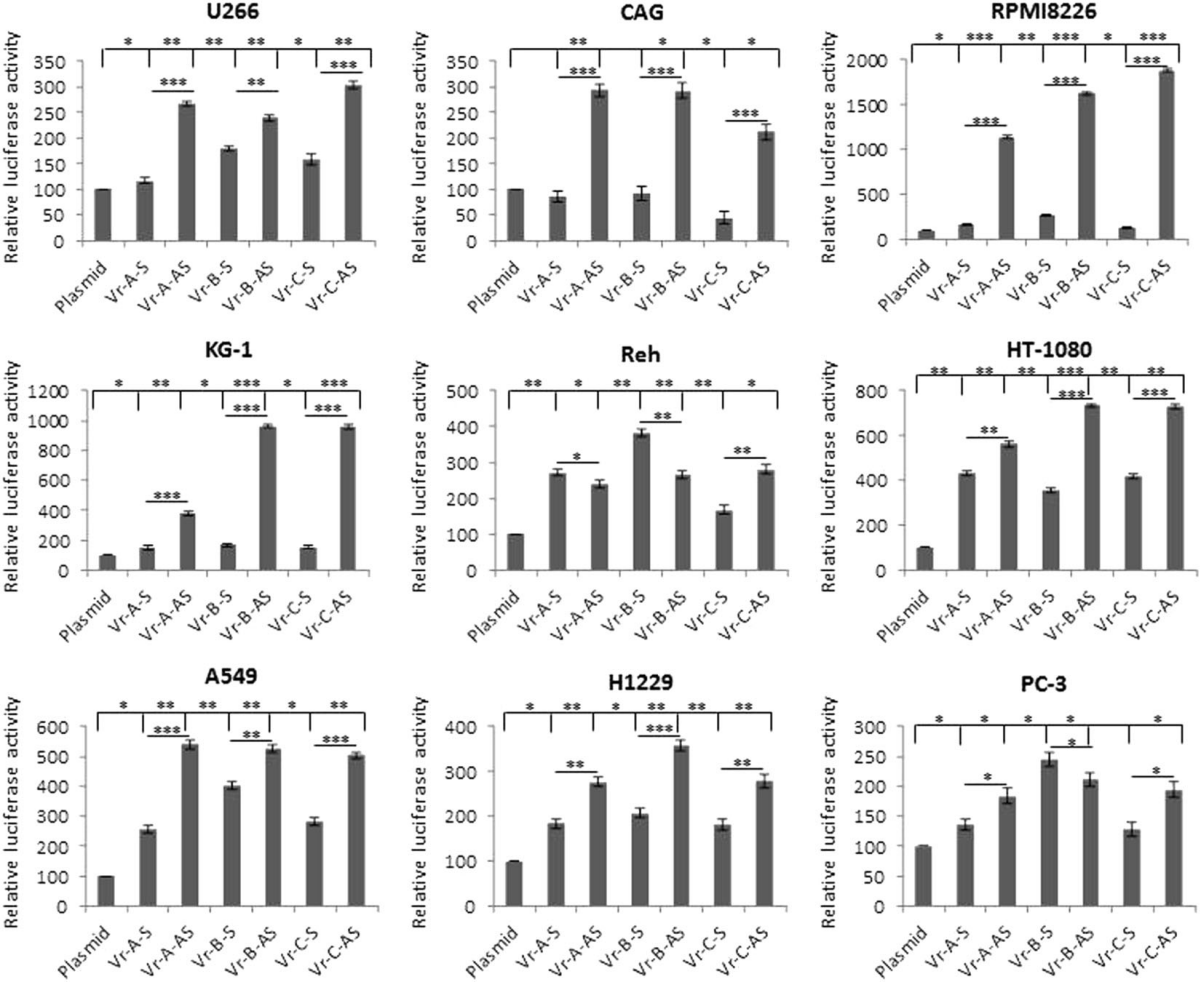
Reporter activity of the HPSE gene enhancer containing various polymorphisms. Six HPSE enhancer constructs were designed and inserted upstream of the luciferase gene in pGL4.26 vector with minimal promoter. The results were derived from five hematological malignancies (U266, CAG, RPMI8226, KG-1, and Reh) and four solid tumor cell lines (HT-1080, A549, H229, and PC-3). Luciferase activity of pGL4.26 without insert was used as a reference (100%). Data shown are of representative experiments performed in triplicates. Differences in luciferase activities among various constructs were evaluated by *t*-test. *p* value of ≤0.05 was regarded as statistically significant. *p value of ≤0.05; **p value of ≤0.001; ***p value of ≤0.0001

To determine the existence of the active enhancer of HPSE in intron 2 and the functional effects of enhancer SNPs we applied the luciferase reporter gene with minimal promoter to measure enhancer activity. Five hematological malignancies (U266, CAG, RPMI8226, KG-1, and Reh) and four solid tumor (HT-1080, A549, H1229, and PC-3) cell lines were transiently transfected with each of the six DNA constructs (Fig. 2) or an empty vector. Our results indicate that the 440 bp fragment of HPSE exhibits enhancer activity in both the sense and antisense directions of the HPSE gene (Fig. 3). Relative luciferase activity was higher in constructs with antisense direction of the enhancer in most of the tested cell lines. Moreover, constructs which included the allele G (Vr-B) revealed elevated levels of luciferase activity compared to constructs which included the A allele (Vr-A) (Table 2). Vr-C is a combination between Vr-A and Vr-B. It also includes the allele G of rs4693608; however, genotypes of additionally linked SNPs (rs4693083, rs4693084, and rs4693609) are identical to Vr-A. Enhancer activity of variant Vr-C was sometimes similar to that of Vr-A, and occasionally to Vr-B, depending on the cell line.

**Table 2:** SNPs effect on the reporter activity of the HPSE gene enhancer

Cell lines	SNP variants (sense)	*p* value	SNP variants (antisense)	*p* value
U226	A–B	0.0003	A–B	0.006
	A–C	0.008	A–C	0.004
	B–C	0.04	B–C	0.0004
CAG	A–B	NS	A–B	NS
	A–C	0.01	A–C	0.002
	B–C	0.01	B–C	0.003
RPMI8226	A–B	0.0003	A–B	5.8 × 10^−6^
	A–C	0.02	A–C	3.8 × 10^−7^
	B–C	0.0001	B–C	0.7 × 10^−5^
KG-1	A–B	NS	A–B	3.1 × 10^−6^
	A–C	NS	A–C	9.1 × 10^−7^
	B–C	NS	B–C	NS
REH	A–B	0.0003	A–B	NS
	A–C	0.0005	A–C	0.02
	B–C	2.48 × 10^−5^	B–C	NS
HT-1080	A–B	0.001	A–B	0.0001
	A–C	NS	A–C	9.98 × 10^−5^
	B–C	0.002	B–C	NS
A459	A–B	0.0001	A–B	NS
	A–C	0.06	A–C	0.03
	B–C	0.0003	B–C	NS
H1229	A–B	0.05	A–B	0.001
	A–C	NS	A–C	NS
	B–C	0.04	B–C	0.004
PC-3	A–B	0.0004	A–B	0.06
	A–C	NS	A–C	NS
	B–C	0.0003	B–C	NS

### The A–G substitution of rs4693608 alters nuclear protein binding to the enhancer region

The nuclear extract of 12 hematological cancer cell lines (Reh, MOLT-3, CEM, Jurkat, U-937, KG-1, K-562, NK-92, NKL, SU-DHL4, CAG, and U266), 14 solid tumor cell lines (A549, H1229, HT-1080, 5637, PC-3, JAR, COLO205, SK_N_SH, PANC-1, MCF-7, HeLa, T84, AGS, and Saos-2), and primary white blood cells from healthy individuals and patients with AML and ALL were incubated with biotin-labeled probes representing the enhancer region of either the A or G alleles and subjected to electrophoretic mobility shift assay. DNA–protein complexes appeared as a band shift pattern. The specificity of the protein–oligonucleotide interaction was shown for both alleles by the reduced intensity of the shifted complex upon addition of unlabeled oligonucleotide probe. Excess unlabeled control oligonucleotide probes compete for binding of the target proteins in control samples.

**Fig. 4:**
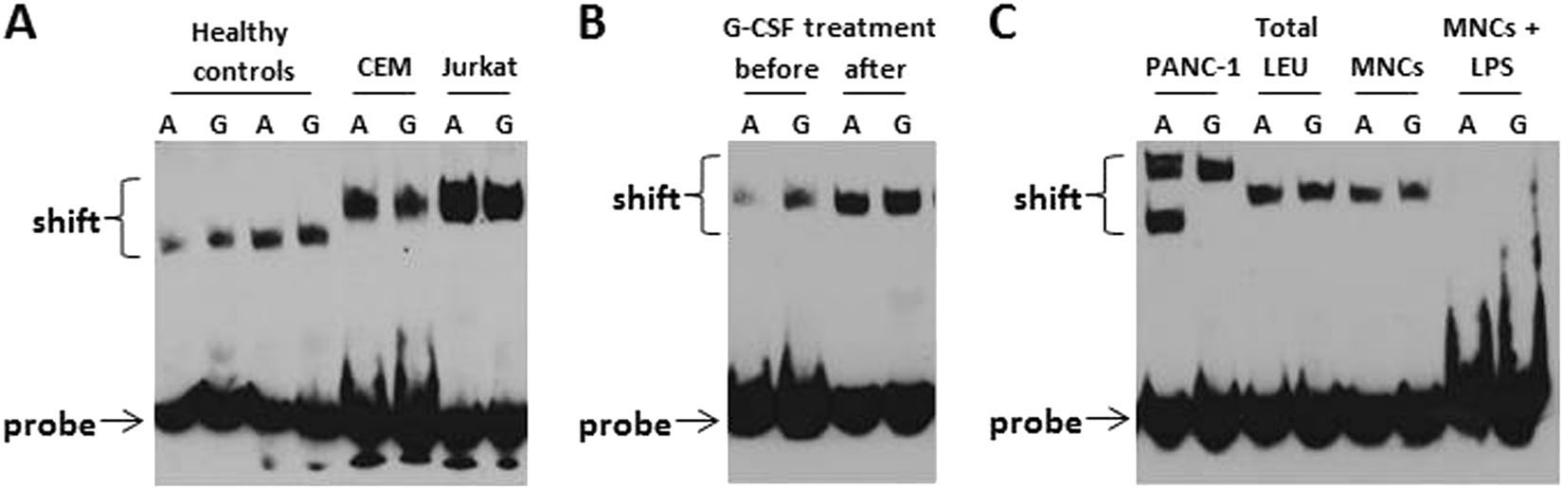
Electromobility shift assay (EMSA) of the rs4693608 SNP using allele-specific oligonucleotide probes in samples of healthy individuals before and after G-SCF and LPS treatment and in comparison to cancer cell lines. Nuclear protein extracts from total leukocytes of healthy donors were incubated with biotin-labeled probes representing the enhancer region and the A or G allele of rs4693608 SNP, and analyzed by electrophoretic mobility shift assay. **a** Analysis of normal samples by EMSA. Gel shift bands were found for both allelic probes. The DNA–protein complexes from normal samples were shifted to a lesser extent in comparison to malignant cell lines. **b** Effect of G-CSF on DNA–protein complex formation. Total leukocytes of healthy donors before and after 5 days G-CSF treatment and before graft collection were obtained. EMSA revealed that G-SCF led to elevation of nuclear proteins, forming DNA/protein complexes in both alleles. **c** Effect of overnight incubation with LPS on DNA/protein interaction. LPS treatment resulted in loss of DNA/protein complexes

**Fig. 5:**
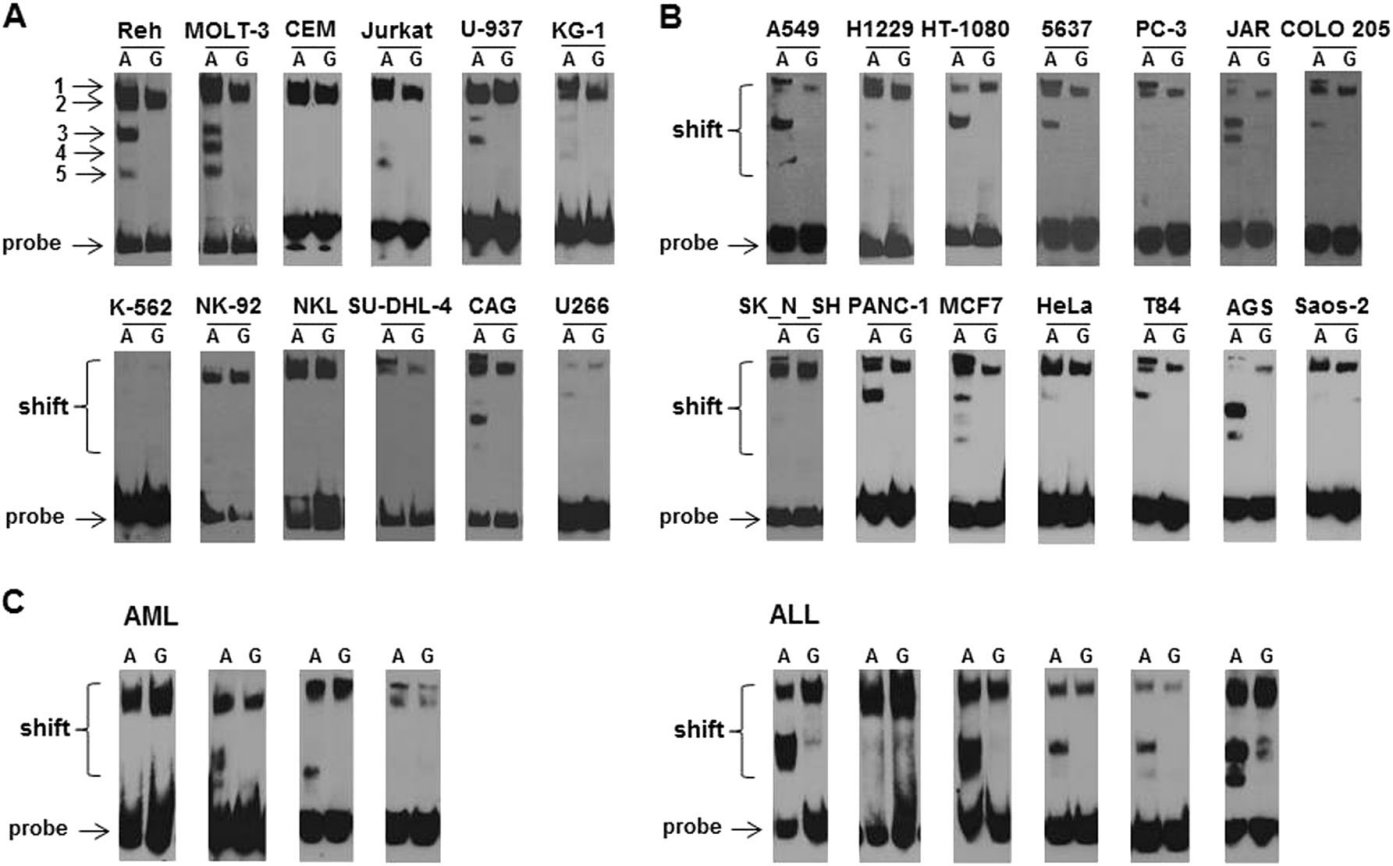
Electromobility shift assay (EMSA) of the rs4693608 SNP using allele-specific oligonucleotide probes in various cell lines and primary patient samples. Nuclear protein extracts from 12 hematological malignant cell lines (**a**), 14 solid tumor cell lines (**b**), and primary samples from patients with acute myeloid (AML) and acute lymphoblastic (ALL) leukemia (**c**) were incubated with biotin-labeled probes representing the enhancer region of either the A or G allele and subjected to electrophoretic mobility shift assay. DNA–protein complexes are reflected by a band shift pattern. The specificity of the protein–oligonucleotide interaction was shown for both alleles by the reduced intensity of the shifted complex upon addition of unlabeled oligonucleotide probe. At least five different variations of DNA–protein complexes bound to allele A were found as compared to only one complex bound to allele G

Analysis of healthy control samples demonstrated gel shift bands for both allelic probes (Fig. 4a). The affinity of the complex to the G allele probe was higher in comparison to the A allele probe. In all analyzed cancer cell lines the band of DNA–protein complex was shifted significantly more in comparison to normal samples (Figs. 4a and 5), likely due to additional proteins recruited to the complex.

EMSA analysis of 26 different cell lines revealed that a single DNA/protein complex binds to allele G, while multiple complexes bind to allele A (Fig. 5a, b). At least five different shifted bands were detected after incubation with allele A probe, reflecting the complexity of the DNA/protein interactions. Some of the cell lines exhibited the same shifted bands pattern while other revealed individual shifted bands (Fig. 5a, b). Analysis of AML and ALL patient samples disclosed a pattern of shifted bands similar to cancer cell lines, but the affinity of the DNA/protein complexes was significantly higher in the majority of the leukemia samples (Fig. 5c).

In addition, we analyzed the effect of G-CSF and LPS on the DNA/protein complexes. Nuclear protein extracts from total leukocytes of healthy donors before and after 5 days of G-CSF treatment and prior to graft collection were obtained. EMSA revealed that G-SCF led to an elevation of nuclear protein levels including the amount of DNA/protein complexes for both alleles (Fig. 4b). In contrast, LPS treatment resulted in disappearance of DNA/protein complexes (Fig. 4c). No difference was found in affinity and shifted bands pattern between DNA/proteins complexes in normal total leukocytes versus normal mononuclear cells (MNCs)^28^.

### Analysis of nuclear protein binding to the DNA in the enhancer region containing rs4693084, rs4693083, and rs4693609 SNPs

**Fig. 6:**
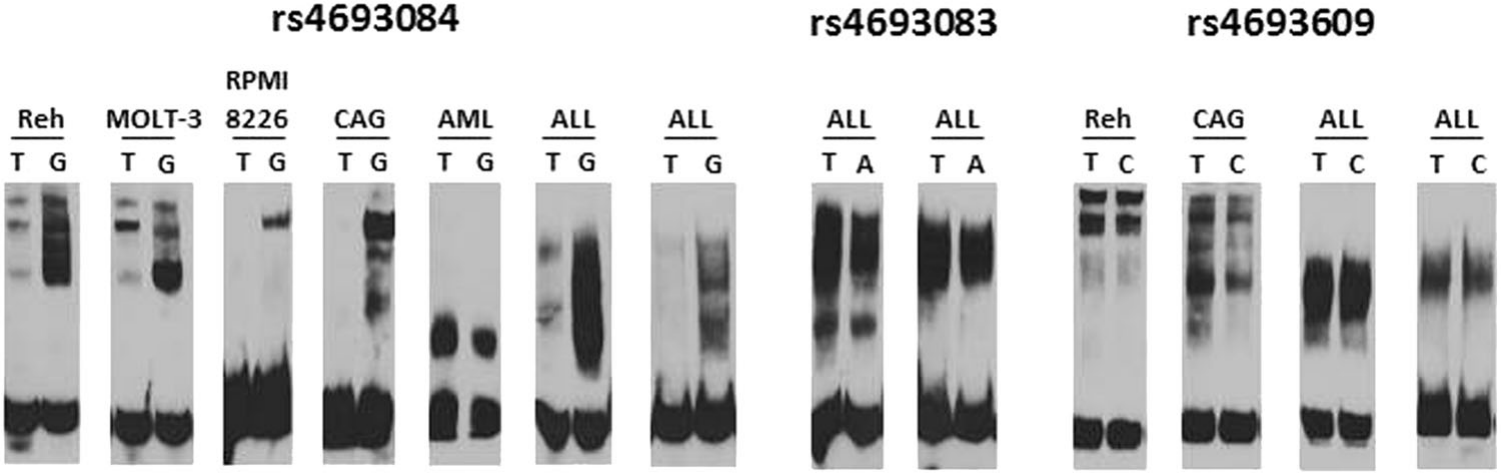
Electromobility shift assay (EMSA) of rs4693084, rs4693083, and rs4693609 SNPs using allele-specific oligonucleotide probes in various cell lines and primary patient samples. Nuclear protein extracts from hematological cell lines and AML/ALL primary samples were incubated with three allele-specific biotin-labeled probes. **a** EMSA for rs4693084, **b** EMSA for rs4693083, and **c** EMSA for rs4693609. Note that only rs4693084 SNP affects proteins binding to DNA in the SNP region of both cell lines and primary patient samples. No differences in protein binding between alleles of rs4693083 and rs4693609 SNPs were detected

The distance between rs4693608 and the other three SNPs is short. Therefore, these SNPs together with rs4693608 may be part of a large DNA/protein complex. The effect of additional three SNPs was analyzed. For this purpose, three allele-specific biotin-labeled probes were generated and EMSA was performed in hematological malignant cell lines and leukemia samples. The assay revealed that only rs4693084 affected protein binding to DNA in the SNP region in both the cell lines and primary patient samples. In multiple myeloma (MM) cell lines, DNA/protein complex formation was observed only for the G allele. Analysis of ALL cell lines and primary samples demonstrated gel shifted bands for both allelic probes. The affinity of the complex to the G allele probe was higher in comparison to the A allele probe (Fig. 6). No nuclear protein binding was found with a probe that included rs4693083 in MM and ALL cell lines. No differences in protein binding between alleles of rs4693083 and rs4693609 were detected (Fig. 6).

### Identification of proteins that bind to the enhancer region of HPSE in healthy individuals and cancer cell lines

**Fig. 7:**
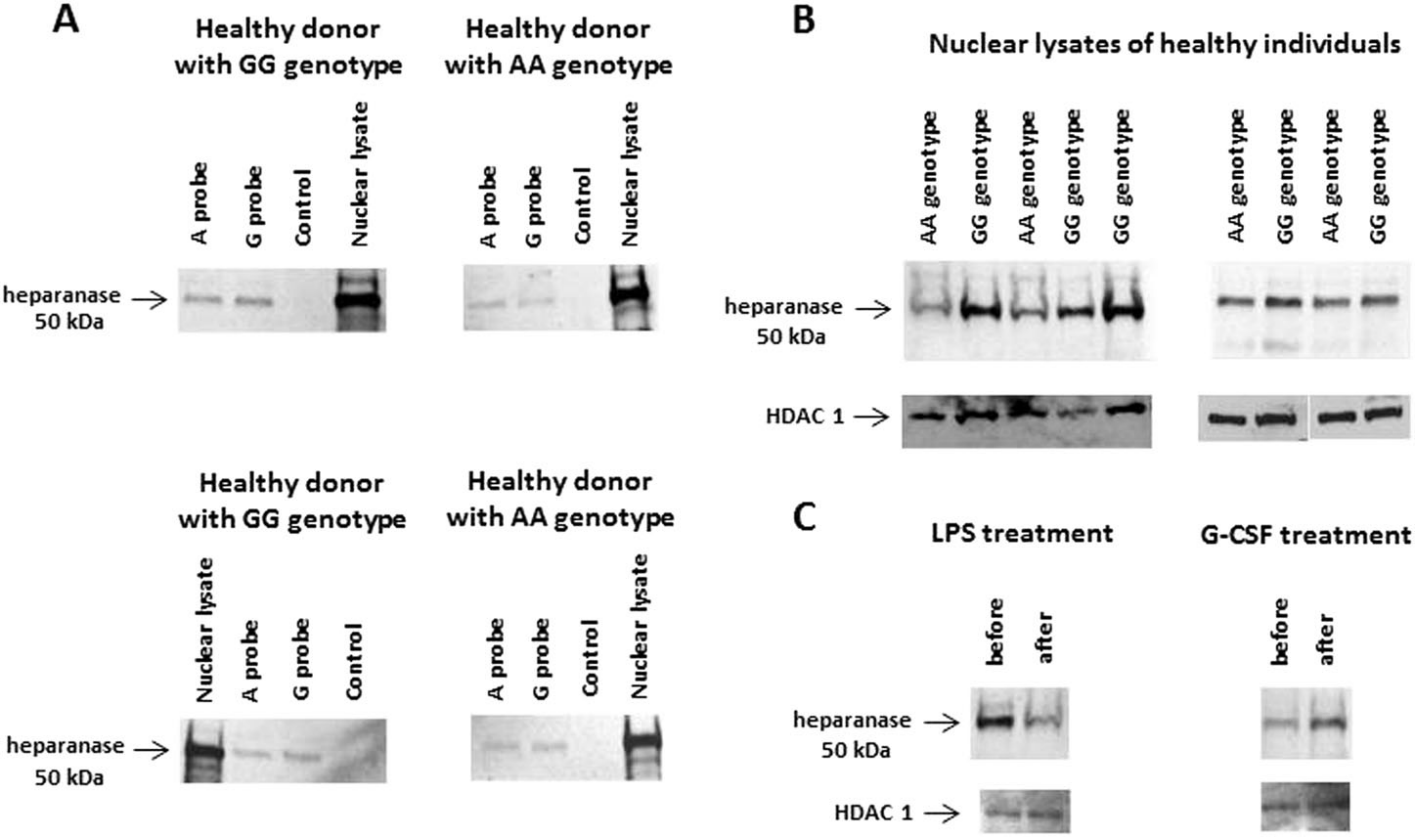
Western blot verification of DNA pull-down assay results in healthy persons. **a** Nuclear protein extracts from total leukocytes of four healthy persons were incubated with M-280 Streptavidin Dynabeads and biotin-labeled probes representing the enhancer region and the A or G allele of rs4693608 SNP and analyzed by western blot (WB) using antibody-specific to heparanase. Protein size of active heparanase (50 kDa) was determined according to molecular weight marker. **b** WB analysis of nuclear lysates extracted from nine normal persons with opposite AA and GG genotypes using anti-heparanase antibody. Heparanase protein levels were higher in the persons with GG genotype in comparison to possessors of AA genotype. **c** Effect of overnight incubation with LPS and of G-CSF administration on nuclear heparanase level. Nuclear protein extracts from total leukocytes of healthy donors before and after 5 days of G-CSF treatment and prior to graft collection were obtained. While LPS treatment resulted in decreased levels of active heparanase, administration of G-SCF led to increased nuclear heparanase level

**Fig. 8:**
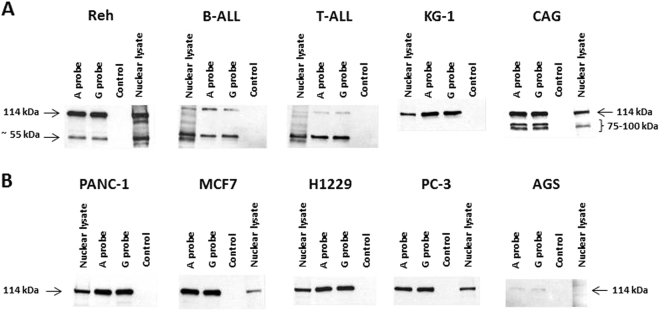
Western blot verification of the DNA pull-down assay in malignant cell lines and primary ALL samples. Nuclear protein extracts from cancer cell lines and primary ALL samples were incubated with M-280 Streptavidin Dynabeads and biotin-labeled probes representing the enhancer region and the A or G alleles of rs4693608 SNP and then analyzed by western blotting (WB) using rabbit-anti-HLTF antibody. Protein size of helicase-like transcription factor (114 kDa) was determined according to molecular weight markers. **a** WB of DNA pull-down products of hematological cell lines and ALL primary samples. **b** WB of DNA pull-down products of solid tumor cell lines

We applied DNA pull-down assay to identify proteins that were bound to the enhancer region. 5′-biotinylated A and G probes that included the rs4693608 SNP were incubated with M-280 Streptavidin Dynabeads and with nuclear extracts from total leukocytes of healthy individuals, Reh, H1229, and PC-3 cell lines. Specific proteins bound to the probes were separated by gradient Mini-PROTEAN^®^ TGX^TM^ Gels and compared with those processed without biotinylated probes. Unique proteins present in the samples were analyzed by mass spectrometry, taking into account the EMSA results. In normal samples we selected proteins that bind to both the A and G alleles but did not appear in control samples and the three cancer cell lines. In contrast, for analysis of malignant cells we chose proteins that were bound to both probes in the three cancer cell lines but were not detected in control samples and normal leukocytes (Supplementary Tables 1 and 2). Proteins that were discovered in the DNA pull-down assay were verified by western blot analyses (Figs. 7 and 8).

Mass spectrometry results of normal samples (Supplementary Table 1) revealed that heparanase itself may bind to the enhancer region. Western blot analysis confirmed this observation (Fig. 7a). Heparanase binds to both A and G probes in the DNA–protein complex. Moreover, the amount of heparanase bound to the G probe was higher than that bound to the A probe. This corresponds to the EMSA results in which the affinity of the complex to the G allele probe was higher in comparison to the A allele probe (Fig. 4). Western blot analysis of nuclear extracts from healthy individuals with AA and GG genotypes showed that heparanase levels were higher in individuals with GG genotype in comparison to possessors of the AA genotype (Fig. 7b). Nuclear heparanase appeared as a 50 kDa protein corresponding to the active form of the enzyme.

Western blot analysis of MNCs pre-treated with LPS or G-CSF revealed a decrease in the amount of nuclear heparanase in response to LPS treatment as opposed to increased level of nuclear heparanase following treatment with G-CSF (Fig. 7c). These observations coincide with the EMSA results (Fig. 4b, c).

Unlike the above results, DNA pull-down analysis followed by western blot verification revealed that heparanase was not detected in the nuclear fraction of malignant cell lines and primary ALL samples. Instead, the helicase-like transcription factor binds to the complex (Supplementary Table 2). According to the EMSA results (Fig. 5), the protein that was bound to the A and G alleles is of molecular weight (MW) higher than that of heparanase. Notably, the MW of HLTF is 114 kDa and it binds to both alleles in each of the malignant samples. Whereas in solid tumor cell lines (PANC I, PC-3, H1229, MCF-7, and AGS), one specific 114 kDa band was noted (Fig. 8b), in hematological cell lines and primary ALL samples additional specific bands were observed (Fig. 8a). It is conceivable that alternative splice forms of HLTF may also bind to this enhancer region. Notably, the amount of HLTF protein that was bound to the enhancer probe in AGS cells line was low (Fig. 8b), corresponding to the EMSA results in this cell line (Fig. 5b).

## Discussion

SNPs located in gene regulatory elements often facilitate the progression of diverse human diseases. Genetic variations contribute to disease development by alterations in transcription factor binding, enhancer activity, long-range enhancer–promoter interactions, posttranslational histone modifications, and/or RNA polymerase function. Identification of causality between non-coding genetic variants, gene regulation, and disease progression and outcome is essential for the development of novel pathways and therapies for disease treatment^29^.

The main objective of our study was to understand how rs4693608 affects heparanase expression and thereby the development of various pathological processes. Using the UCSC Genome Browser website, we identified a potential strong enhancer in the rs4693608 region and then demonstrated that this region exhibits SNPs-dependent enhancer activity. Analysis of normal blood samples revealed binding of DNA/protein complex to both alleles with higher affinity to allele G. All the examined malignant cell lines and primary leukemia samples disclosed a more prominent shift of the main bands in comparison to normal samples. This result is likely due to formation of DNA/protein complexes with additional proteins that were bound to the probes. At least five extra shifted bands were incorporated to allele A probe while allele G probe was bound to only one main DNA/protein complex. Despite a common band pattern, each malignant sample revealed specific shifted bands.

**Fig. 9:**
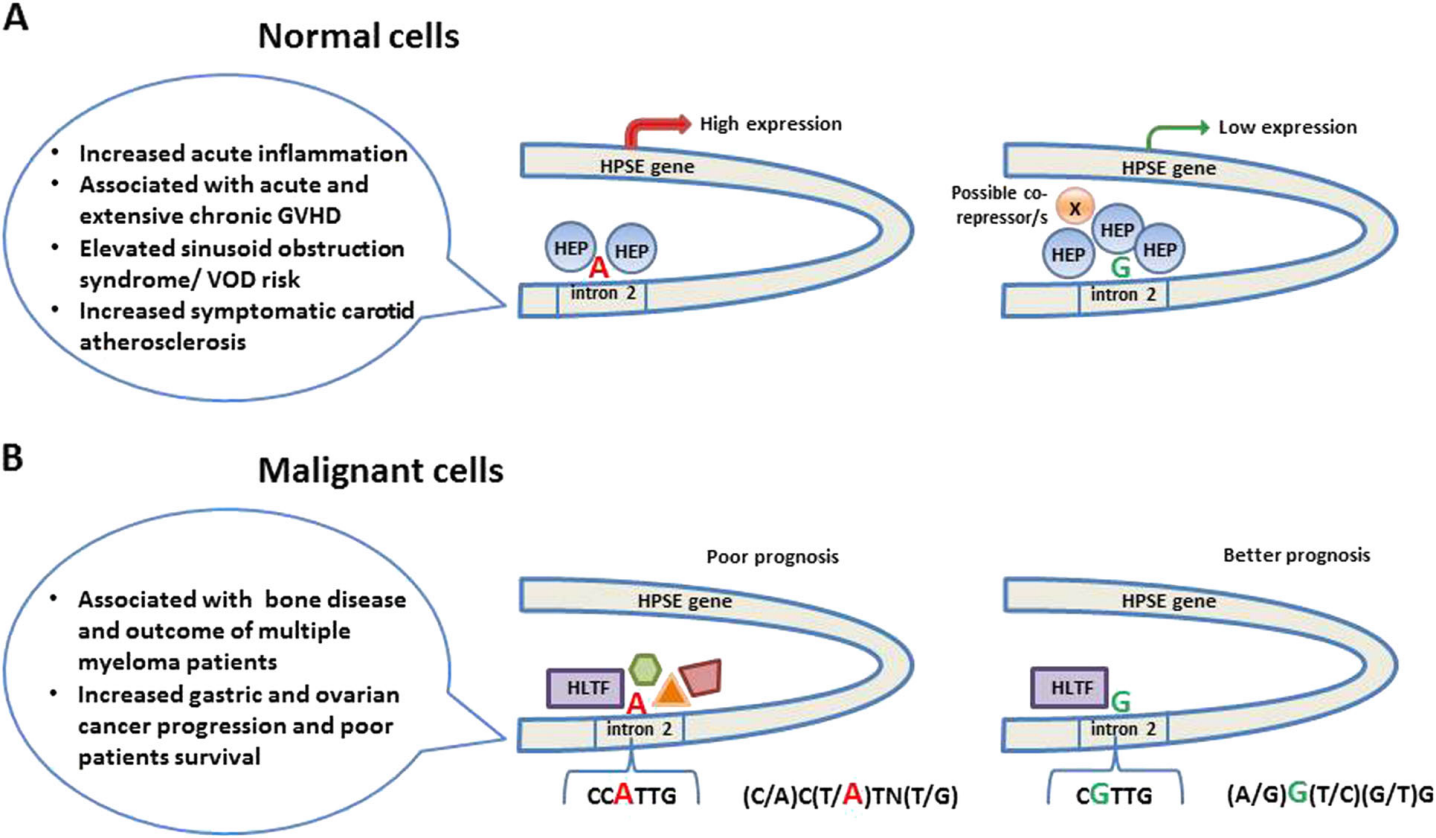
A model of the HPSE gene enhancer regulation in normal and malignant cells. **a** Normal cells. Heparanase binds to the intron 2 enhancer region and regulates HPSE expression by negative feedback in rs4693608 SNP-dependent manner. More molecules of heparanase bind to the enhancer region in carriers of allele G in comparison to possessors of allele A. As a result the expression level of the HPSE gene will be higher in persons with genotype AA than in individuals with genotype GG. Additional co-repressor(s) may be recruited to the complex and further suppress the expression of the HPSE gene. **b** Malignant cells. In malignant cells and primary leukemia samples, heparanase is not recruited to the enhancer region and hence its negative self-regulation is discontinued. Instead, the helicase-like transcription factor (HLTF) binds to the regulatory enhancer region. Two different consensus sequences that are recognized by HLTF were identified in the enhancer region: (C/A)C(T/A)TN(T/G) for allele A and (A/G)G(T/C)(G/T)G for allele G. While in carriers of allele A, HLTF binds to the enhancer region together with other proteins, possessors of allele G bind the HLTF alone. Allele A is associated with cancer progression and poor patient survival in comparison to allele G, which was found in correlation to better disease outcome. It is possibly due to SNP-dependent different levels of heparanase. Identification of downstream mediators in both variations of the HPSE gene will clarify the importance of this newly identified regulatory region in cancer development and progression

DNA pull-down assay followed by western blot verification showed that heparanase binds to the enhancer region of intron 2 and regulates HPSE gene expression via negative feedback in rs4693608 SNP-dependent manner (Fig. 9). This may explain our previously published results of inverse correlation between HPSE gene SNPs and heparanase expression^19^. Thus, an inverse correlation between HPSE mRNA and plasma heparanase levels was associated with the same SNPs. For example, the rare genotype GG of rs4693608 was associated with low HPSE mRNA expression level and high plasma levels of active heparanase. In contrast, the frequent AA genotype of this SNP was associated with high HPSE mRNA expression and low level of plasma heparanase^19^. The present western blot analyses revealed that the level of nuclear heparanase was higher in possessors of the GG genotype in comparison to carriers of the AA genotype (Fig. 7b).

Our previously published study showed that HPSE gene expression was elevated after LPS treatment in both cord blood and adult MNCs in rs4693608 SNP-dependent manner^21^. Our present EMSA and western blot results obtained with LPS-treated MNCs support such a negative feedback regulation of heparanase expression. LPS treatment led to disappearance of DNA/protein complexes and a decrease in nuclear heparanase. Further research is needed to clarify how A–G substitution influences on a negative feedback of heparanase regulation. We assume that additional co-repressor(s) may take part in this regulation and that binding of heparanase, in *cis* or *trans*, to the enhancer region could explain the effect of rs4693608 SNP on HPSE gene expression. These results are important to better understand the involvement of heparanase and rs4693608 SNP in acute inflammation, acute and extensive chronic GVHD, risk of sinusoid obstruction syndrome/VOD following allogeneic stem cell transplantation, and symptomatic carotid atherosclerosis (Fig. 9)^20–23^.

Our results revealed that in malignant cell lines and primary leukemia samples, heparanase discontinues self-regulation of the enhancer region. Instead of heparanase, the helicase-like transcription factor (HLTF) binds to the regulatory region. HLTF is a member of the yeast mating SWItch/sucrose non-fermenting (SWI/SNF) family of proteins involved in chromatin remodeling. HLTF plays a significant role in gene transcription, DNA repair, ubiquitination, and support of genome stability^30,31^. HLTF appears to function as a tumor suppressor, supported by the detection of HLTF promoter hypermethylation in cancer tissues and cell lines^31,32^. Recent studies, however, reveal increased expression of HLTF in transformed cells and cancer specimens, suggesting that HLTF could be associated with carcinogenesis and may function as an oncogene^32^. Two different consensus sequences recognized by HLTF were proposed: (C/A)C(T/A)TN(T/G) and (A/G)G(T/C)(G/T)G^30^. Analysis of the HPSE enhancer region included in rs4693608 SNP indicated that in the case of allele A, HLTF recognizes the first sequence CC**A**TTG. A–G alteration does not result in loss of the transcription site but rather a change to the second variant of the HLTF recognition site (C**G**TTG), except the first nucleotide. Further studies will clarify if these recognition sites of HLTF may activate various downstream processes. Our EMSA results disclosed that not only HLTF binds to the HPSE enhancer region in allele A, but other proteins may form a common DNA–protein complex. In the case of allele G only HLTF binds to the HPSE gene enhancer region (Fig. 9). It is therefore important to understand how different types of DNA–protein binding complexes influence heparanase expression and affect its involvement in cancer progression. Future identification of particular proteins bound to allele A of rs4693608 will likely reveal proteins specific to each cell type and disease situation. ChIP analysis applying specific ChIP-graded antibodies will enable to further elucidate the mode of interaction between DNA and proteins, involved in regulating the HPSE intron enhancer in specific cells and processes.

Enhancers are regulatory DNA sequences widely scattered throughout the genome and involved in gene activation independent of their orientation or distance. Enhancers determine the precise tissue-specific gene expression pattern during normal development and are regarded as key players in directing disease transcriptional program during pathogenesis^33,34^.

According to the UCSC Genome Browser website, HPSE enhancer region reveals all signs of active enhancer. Histone H3 lysine 4 monomethylation (H3K4me1), dimethylation (H3K4me2), and histone H3 lysine 27 acetylation (H3K27ac) sites were detected around rs4693608 in K-562 cell line, CD14+ and CD20+ immune cells, endothelial cells (Huvec), and adult normal human dermal fibroblasts (NHDF-Ad).

Our previous study revealed strong correlation between rs4693608 and heparanase expression among healthy individuals^19^. The present study indicates that this is likely mediated by the enhancer activity. In a previously published study, Parish et al. demonstrated a novel role for nuclear heparanase as a direct epigenetic mediator of histone H3 methylation across immune responsive genes in human T cells^13,16^. Heparanase binds to chromatin and acts as part of an active multi-subunit complex, which includes RNA polymerase II and the lysine-specific demethylase 1 (LSD1). This complex was identified in the promoter and 5′ transcribed regions of inducible immune genes. In addition, heparanase is associated with differentiation and development of activated T cells and with genes affect metabolic and biosynthetic processes in resting T cells. Chromatin-anchored heparanase also targets a distinct cohort of microRNA promoters previously found to play a key role in T cell functions^13,16^. It is not clear if the newly identified enhancer affects expression of other genes. An indirect epigenetic effect of rs4693608 and enhancer activity on heparanase function is a likely possibility.

According to MeDIP-seq (methylated DNA immunoprecipitation and sequencing) of the UCSC Genome Browser and MethPrimer prediction tools, allele G of rs4693608 is part of a putative CpG island. G–A alteration leads to loss of the CpG site. CpG islands are the basis of DNA modifications such as methylation and hydroxymethylation. In mammals, the majority of cytosines (70–80%) in CpG dinucleotides are methylated in somatic cells. DNA methylation has been evidently linked to transcriptional regulations. Numerous studies focus on differentially methylated regions in complex diseases. The role of DNA methylation in cancer etiology and progression is well established^13,35^. Our EMSA results indicate that while allele G of rs4693608 is susceptible to DNA methylation, allele A may escape this modification. Thus, at least five different shifted bands were found after incubation with allele A probe in various malignant cell lines and leukemia primary samples, only one main shifted band was detected after incubation with allele G probe.

In our study we analyzed three additional SNPs located in the enhancer. Only rs4693084 affected protein binding to DNA in the SNP region of cell lines and leukemia primary samples. In addition, all three SNPs were found in strong LD with rs11099592. Previous studies revealed association between rs11099592 and the risk of ALL development. Additional correlation was found with the outcome of gastric cancer^18,24,25^. Given the strong LD between rs11099592 and rs4693084, disease associations may result from different enhancer activity. The distance between rs4693608 and rs4693084 is only 17 bp, and under appropriate conditions both SNPs may be part of a common DNA/protein complex.

Analysis of the functional effect of SNPs on luciferase activity revealed that constructs that included the G allele (Vr-B) exhibited higher levels of luciferase in comparison to constructs which included the A allele. Taken into account the EMSA and western blot results in malignant cells, we assume that the activity of HPSE enhancer depends on the quality and quantity of DNA/protein complexes. When the number of nuclear proteins bound to the enhancer is low, the enhancer activity is high.

Remarkably, SNPs appear to affect binding of transcription factors to the enhancers and thereby modulate the expression of target genes. Importantly, regions termed variant enhancer loci (VEL) regulate the complex interactions between genetic and epigenetic elements that redefine which enhancers are turned on or off in cancer^36,37^.

The present study identified the HPSE gene enhancer, located in intron 2, which is involved in self-regulation of heparanase by a negative feedback exerted in rs4693608-dependent manner. During carcinogenesis, self-regulation of heparanase is attenuated and the helicase-like transcription factor starts to regulate this enhancer region, again in rs4693608-dependent manner. Analysis of disease-specific cell lines or primary samples allows precise identification of tissue-specific proteins that bind to the HPSE enhancer region. This knowledge could potentially enable modification of the enhancer activity in a way that will modulate the expression of HPSE and thereby influence downstream processes and disease progression.

## Materials and methods

### Samples and cell lines

Human total leukocytes from healthy individuals and patients with AML and ALL as well as malignant cell lines were applied in the study. All subjects gave their written informed consent. Thirteen hematological malignant cell lines (K-562, KG-1, U-937, Reh, MOLT-3, Jurkat, CEM, SU-DHL4, CAG, RPMI8226, U266, NK-92, and NKL) and 15 solid tumor cell lines (HT-1080, JAR, A549, H1229, PC-3, 5637, SK_N_SH, MCF-7, HeLa, COLO205, T84, AGS, Saos-2, PANC-1, and MDA-MB435) were analyzed. The cells were maintained in RPMI1640 and DMEM media (Gibco by Life Technologies, Inc., Paisley, UK) supplemented with 10% fetal bovine serum (FBS), 1 mM l-glutamine, 100 U/ml penicillin, and 0.01 mg/ml streptomycin (Biological Industries, Israel) in a humidified atmosphere of 5% CO_2_ at 37 °C. All cell lines were analyzed by STR DNA fingerprinting using AmpFISTR Identifier Kit (Applied Biosystem) and tested for mycoplasma contamination.

### SNPs analysis of the enhancer region

The enhancer region includes three additional polymorphic SNPs besides rs4693608. Genotypes of rs4693084, rs4693609, rs4693608, and rs11099592 were performed by allele-specific amplification. PCR-RFLPs-based assay was used for rs4693083 detection. The PCR fragments were amplified from genomic DNA (the Wizard^®^ Genomic DNA Purification kit (Promega, Madison, WI), using the forward and reverse primers (Table 3). The PCR reactions were performed as described^17–19^. Annealing temperature was 56 °C for 45 s for rs4693083 and rs4693609, 52 °C for 30 s for rs4693084. Genotyping of rs4693083 was performed by digestion of PCR fragments with *Hph*I restriction endonuclease (New England Biolabs).

**Table 3:** Primer sequences for SNPs detection and generation of allele-specific probes

Name of SNP or probe	Modification	Sequence of primers	PCR/probe product size, bp
rs4693083 SNP	T → A	Forward: 5′ CTGGGCGACAAAGCGAGACA 3′	285
		Reverse: 5′ TGACCAGGGTGGATTTTTTC 3′	
rs4693084 SNP	T → G	T allele forward: 5′ TTGACTTTTTTTCGCTTT 3′	238
		G allele forward: 5′ TTGACTTTTTTTCGCTTG 3′	
		Reverse: 5′ ACAGGCACACACCACCACG 3′	
rs4693609 SNP	C → T	Forward: 5′ TGTTTTCCTCTTGCCATCAT 3′	279
		T allele reverse: 5′ CACACACCACCACGCCCAG 3′	
		G allele reverse: 5′ CACACACCACCACGCCCAA 3′	
Enhancer cloning		Forward: 5′ CTGGGCGACAAAGCGAGACA 3′	440
		Reverse: 5′ ACAGGCACACACCACCACG 3′	
rs4693608 probe	A → G	A allele forward: 5′ [Btn]GGGTGGGAACC**A**TTGACTTTTTTTCGC 3′	30
		A allele reverse: 5′ [Btn]GCGAAAAAAAGTCAA**T**GGTTCCCACCC 3′	
		G allele forward: 5′ [Btn]GGGTGGGAACC**G**TTGACTTTTTTTCGC 3′	30
		G allele reverse: 5′ [Btn]GCGAAAAAAAGTCAA**C**GGTTCCCACCC 3′	
rs4693083 probe	T → A	T allele forward: 5′ [Btn]TCTGCAGTGTGGTG**T**TTTCCTCTTGCCATC 3′	30
		T allele reverse: 5′ [Btn]GATGGCAAGAGGAAA**A**CACCACACTGCAGA 3′	
		A allele forward: 5′ [Btn]TCTGCAGTGTGGTG**A**TTTCCTCTTGCCATC 3′	30
		A allele reverse: 5′ [Btn]GATGGCAAGAGGAAA**T**CACCACACTGCAGA 3′	
rs4693084 probe	T → G	T allele forward: 5′ [Btn]ACTTTTTTTCGCTT**T**GAATTTGGTACCATC 3′	30
		T allele reverse: 5′ [Btn]GATGGTACCAAATTC**A**AAGCGAAAAAAAGT 3′	
		G allele forward: 5′ [Btn]ACTTTTTTTCGCTT**G**GAATTTGGTACCATC 3′	30
		G allele reverse: 5′ [Btn] GATGGTACCAAATTC**C**AAGCGAAAAAAAGT 3′	
rs4693609 probe	C → T	C allele forward: 5′ [Btn]TACGAAAACTTAG**C**TGGGCGTGGTGGTGT 3′	30
		C allele reverse: 5′ [Btn]ACACCACCACGCCCA**G**CTAAGTTTTCGTA 3′	
		T allele forward: 5′ [Btn]TACGAAAACTTAG**T**TGGGCGTGGTGGTGT 3′	30
		T allele reverse: 5′ [Btn]ACACCACCACGCCCA**T**CTAAGTTTTCGTA 3′	

### DNA constructs

The HPSE gene fragment of intron 2 (440 bp), which includes rs4693083, rs4693608, rs4693084, and rs4693609 SNPs, was cloned via PCR using PCR II-TOPO vector (Invitrogen by Life Technologies, Carlsbad, CA). The forward and reverse primers for PCR amplification of the enhancer region are shown in Table 1. The enhancer fragments were digested with *Hin*dIII and *Xho*I and ligated into phosphatase-treated pGL4.26 (luc2/minP/Hygro) vector (Promega, Madison, WI), which was digested with the same enzymes. JM109 competent cells (Promega, Madison, WI) were used for cloning. Six DNA constructs were prepared, three in sense direction and three in antisense direction (Fig. 2). The direction of all constructs was confirmed by DNA sequencing.

### Luciferase reporter assay

All cell lines (U266, CAG, RPMI8226, KG-1, Reh, HT-1080, A549, H1229, and PC-3) were seeded in six-well culture dishes according to Lonza instruction (AMAXA Biosystems, Lonza, Germany). For the luciferase assay, cells were transfected with 2 µg of each DNA contract using the Ingenio Electroporation Kit (Mirus Bio, Madison, WI) and Nucleofector^TM^ (AMAXA biosystems, Lonza, Germany).

Twenty-four hours after electroporation, the cells were lysed in passive lysis buffer (Promega, Madison, WI). The luciferase assay system (Promega, Madison, WI) and 20/20^n^ Luminometer (Turner BioSystems, Sunnyvale, CA) were used for the firefly luciferase activity measurement. Two methods were applied for normalization. The first was total protein quantification according to the Bradford method (Bio-Rad, Hercules, CA). pGL4.73 (*hRluc*/SV40) vector was used as an internal control vector (Promega, Madison, WI) in the second method. Luciferase activity of pGL4.26 without insert was defined as a 100%. The data shown are representative examples of at least three experiments, which were performed in triplicates. Differences in luciferase activities among constructs were determined by *t*-test. A *p* value of ≤0.05 was considered as statistically significant.

### Electromobility shift assay (EMSA)

The primer sequences used for generating allele-specific biotin-labeled probes are shown in Table 3. Altered bases are marked in bold. Oligonucleotide probes were generated by heating the complementary oligonucleotides in annealing buffer (10 mM Tris, pH 7.5–8.0, 50 mM NaCl, 1 mM EDTA) at 95 °C for 5 min and then slowly cooled to room temperature. Nuclear protein extracts derived from cell lines and primary normal and leukemia samples were obtained using a Nuclear Extraction Kit (Millipore, Temecula, CA). EMSA was performed using Gelshift Chemiluminescent EMSA Kit (Active Motif, Rixensart, Belgium) according to the manufacturer’s protocol. Twenty fmol of biotin-labeled oligonucleotides were incubated for 20 min with nuclear extracts (8 µg). Competitive binding was performed by including 4 pmol unlabeled oligonucleotides in control reactions. Biotin-labeled oligonucleotide-nuclear extract complexes were separated by 6% non-denaturing polyacrylamide gel in 0.5× TBE cooled buffer, at 100 V for 1.5 h and transferred onto positively charged nylon membranes BrightStar-Plus (Ambion by Life Technologies, Austin, TX) by Mini-Trans-Blot Electrophoretic Transfer Cell. Blots were visualized by chemiluminescence. The data shown are representative examples of at least three experiments

### Pull-down assay

The pull-down assay was performed using Dynabeads M-280 Streptavidin (Invitrogene by Thermo Fisher Scientific, Norway). An aliquot of 20 µl of beads was washed in PBS-T (0.1% Triton X-100) and incubated with 500 ng of each biotinylated DNA probe overnight at 4 °C. The beads were washed in PBS-T and then in pull-down wash buffer containing 20% glycerol, 0.1 M KCl, 0.2 mM EDTA, 20 mM Hepes-KOH pH 7.9, 1 mM DTT, 0.1% Triton X-100, 0.1× Halt Protease inhibitor Cocktail (Thermo Scientifec, Rockford, IL, USA), 0.1 mM PMSF. Nuclear protein extract (500 µg) was incubated with beads for 15 min at 4 °C in pull-down incubation buffer (5% glycerol, 150 mM KCl, 0.05 mM EDTA, 0.025% Triton X-100, 5 mM Hepes-KOH pH 7.9, 1 mM DTT, 0.1× Halt Protease inhibitor Cocktail, 0.1 mM PMSF). The beads were washed in pull-down wash buffer, eluted in sample buffer, and boiled at 95 °C for 5 min.

### Mass spectrometry

Proteins, which were eluted by the DNA pull-down assay, were run into a Mini-PROTEAN^®^ TGX^TM^ 4%-20% precast Gels (BIO-RAD Laboratories, Inc. USA) and stained with Imperial^TM^ Protein Stain (Thermo Scientific, Rockford, IL, USA). The stained gel pieces were digested with trypsin, and then analyzed by LC-MS/MS on Q-Exactive plus (Thermo) and identified by Discoverer software (Discoverer 1.4) with two search algorithms: Sequest (Thermo) and Mascot (Matrix Science) against the human proteome from the Uniprot database, and a decoy database. All the identified peptides were filtered with high confidence, top rank, mass accuracy, and a minimum of two peptides. High-confidence peptides have passed the 1% FDR (false discovery rate) threshold. Semi quantitation was done by calculating the peak area of each peptide. The area of the protein is the average of the three most intense peptides from each protein.

### Western blotting

Pulled down proteins were separated on Mini-PROTEAN^®^ TGX^TM^ 4–20% precast gels (BIO-RAD Laboratories, Inc. USA) and then transferred onto a 0.2 µm nitrocellulose membrane by Trans-Blot^®^ Turbo^TM^ Transfer Pack (BIO-RAD Laboratories, Inc. USA) and Transfer Blot Turbo Transfer System (BIO-RAD). Each membrane was blocked with 5% BSA in PBS-T. A rabbit-anti-HLTF antibody (Abcam, ab17984) and rabbit-anti-heparanase antibody (InSight Pharmaceuticals, Rehovot, Israel) were applied for the assay. After washing, membrane was incubated with HRP-conjugated anti-rabbit secondary antibody. Detection was performed by applying enhanced chemiluminescence (EZ-ECL, Biological Industries, Israel).

## Electronic supplementary material

Supplementary Table 1

Supplementary Table 2
