# Supplementary material for: Identification of strong intron enhancer in the heparanase gene: effect of functional rs4693608 variant on HPSE enhancer activity in hematological and solid malignancies
Source: Oncogenesis. 2018 Jun 29;7(6):51. doi: 10.1038/s41389-018-0060-8 (PMC6023935; doi:10.1038/s41389-018-0060-8)
Supplement: Supplementary file 1 — Supplementary Table 1 [file 41389_2018_60_MOESM1_ESM.docx]

**Supplementary Table1. Mass spectrometry results of DNA pull-down products from normal sample.**

| **№** | **Protein name** | **Normal** | | **H1229** | | **Reh** | | **PC3** | |
| --- | --- | --- | --- | --- | --- | --- | --- | --- | --- |
|  |  | **A** | **G** | **A** | **G** | **A** | **G** | **A** | **G** |
| 1  2  3  4  5  6  7  8  9  10 | Talin-1  **Heparanase**  Compliment C1q  Band 3 anion transport protein  Glycoprotein Ib, α polypeptide  T-complex protein 1 subunits zeta  Abl interactor 1  Beta-arrestin-2  Minor histocompatibility protein HA  Nucleosome assembly protein 1-like 1 | 4.88*10^7^  9.76*10^7^  8.76*10^7^  1.8*10^7^  3.76*10^6^  7.39*10^6^  1.88*10^7^  4.11*10^7^  5.41*10^6^  4.2*10^7^ | 1.47*10^8^  1.04*10^8^  3.66*10^8^  2.46*10^7^  3.32*10^7^  2.23*10^7^  2.81*10^7^  3.35*10^7^  1.06*10^7^  5.03*10^7^ | 0  0  0  0  0  0  0  0  0  0 | 0  0  0  0  0  0  0  0  0  0 | 0  0  0  0  0  0  0  0  0  0 | 0  0  0  0  0  0  0  0  0  0 | 0  0  0  0  0  0  0  0  0  0 | 0  0  0  0  0  0  0  0  0  0 |

The numbers represent the average area of the three unique peptides with the largest peak area.
