# Supplementary material for: Identification of strong intron enhancer in the heparanase gene: effect of functional rs4693608 variant on HPSE enhancer activity in hematological and solid malignancies
Source: Oncogenesis. 2018 Jun 29;7(6):51. doi: 10.1038/s41389-018-0060-8 (PMC6023935; doi:10.1038/s41389-018-0060-8)
Supplement: Supplementary file 2 — Supplementary Table 2 [file 41389_2018_60_MOESM2_ESM.docx]

**Supplementary Table2. Mass spectrometry results of DNA pull-down products from cancer cell lines.**

| **№** | **Protein name** | **H1229** | | **Reh** | | **PC3** | | **Normal** | |
| --- | --- | --- | --- | --- | --- | --- | --- | --- | --- |
|  |  | **A** | **G** | **A** | **G** | **A** | **G** | **A** | **G** |
| 1  2  3  4  5  6  7  8  9  10 | DNA ligase 3  **Helicase-like transcription factor**  DNA repair protein XRCC1  Replication protein A 32kDa subunit  Microtubule-associated protein 4  UV excision repair protein RAD23  DNA-3-methyl adenine glycolase  Replication factor C subunit 5  Centrin-2  Protein Wiz | 5.79*10^9^  2.62*10^9^  5.94*10^9^  5.08*10^8^  1.73*10^7^  4.93*10^8^  1.63*10^8^  6.36*10^7^  5.12*10^8^  9.99*10^6^ | 4.71*10^9^  2.22*10^9^  4.43*10^9^  1.92*10^8^  1.38*10^7^  4.16*10^8^  2.38*10^8^  3.93*10^7^  3.46*10^8^  5.34*10^7^ | 9.59*10^7^  1.02*10^9^  1.79*10^7^  4.19*10^8^  4.23*10^7^  9.51*10^7^  1.08*10^8^  4.25*10^7^  9.4*10^7^  3.12*10^7^ | 6.85*10^7^  9.78*10^8^  5.72*10^6^  1.91*10^8^  4.29*10^7^  8.92*10^7^  8.43*10^7^  1.48*10^7^  1.24*10^8^  1.18*10^7^ | 1.87*10^9^  1.46*10^9^  2.1*10^9^  4.67*10^8^  3.69*10^7^  3.18*10^8^  1.12*10^8^  9.46*10^6^  3.11*10^8^  7.04*10^6^ | 2.31*10^9^  1.75*10^9^  2.33*10^9^  2.07*10^8^  9.68*10^7^  2.42*10^8^  1.01*10^8^  8.02*10^6^  2.73*10^8^  1.03*10^7^ | 0  0  0  0  0  0  0  0  0  0 | 0  0  0  0  0  0  0  0  0  0 |

The numbers represent the average area of the three unique peptides with the largest peak area.
